# New Pollen Morphological Perspectives into *Vernonia* (Compositae—Vernonieae) from Madagascar

**DOI:** 10.3390/plants15121927

**Published:** 2026-06-22

**Authors:** Higor Antonio-Domingues, Benoit Loeuille, Mihajamalala Andotiana Andriamanohera, Isabel Larridon, Morgan Robert Gostel, Ana Rita Giraldes Simões

**Affiliations:** 1 Royal Botanic Gardens, Kew, Richmond, Surrey TW9 3AE, UK; b.loeuille@kew.org (B.L.); ama0130@mavs.uta.edu (M.A.A.); i.larridon@kew.org (I.L.); asimoes@mobot.org (A.R.G.S.); 2 C. E. Moss Herbarium, University of the Witwatersrand, Private Bag 3, WITS, Johannesburg 2050, South Africa; 3 Botanical Research Institute of Texas, Fort Worth, TX 76107, USA; 4 University of Texas at Arlington, Arlington, TX 76019, USA; 5 Morris Arboretum and Gardens of the University of Pennsylvania, Philadelphia, PA 19118, USA; 6 Missouri Botanical Garden, Africa & Madagascar Department, 4344 Shaw Boulevard, St Louis, MO 63110, USA; 7 Institut de Systématique, Évolution, Biodiversité (ISYEB), Muséum National d’Histoire Naturelle, Centre National de la Recherche Scientifique (CNRS), Sorbonne Université, École Pratique des Hautes Études, Université des Antilles, CP 39, 57 rue Cuvier, F-75005 Paris, France; 8 Ghent University, K. L. Ledeganckstraat 35, 9000 Gent, Belgium

**Keywords:** Africa, Asteraceae, Madagascar, mesoaperture, paleotropics, palynology, palynotaxonomy

## Abstract

A comprehensive palynotaxonomic study is presented of species of *Vernonia* from Madagascar, documenting 37 (out of 44) taxa using light microscopy (LM) and scanning electron microscopy (SEM). Statistical analyses were performed to explore patterns of variation in quantitative data between the species. The pollen variation is categorised into two pollen types based on qualitative and quantitative characters, which was supported by Principal Components Analysis. A mesoaperture, a unique apertural structure, is also described for the genus for the first time. Further integrative taxonomic studies focused on *Vernonia* and the Vernonieae tribe, including species from continental Africa and Madagascar, are ongoing and will help to determine whether these characters remain taxonomically informative and whether they could be applied in an evolutionary framework.

## 1. Introduction

The tribe Vernonieae (Asteraceae or Compositae) has long been recognised as one of the most taxonomically challenging lineages within the family [1]. Its complex taxonomic history and unstable generic boundaries have led specialists to refer to Vernonieae informally as the “evil tribe”, reflecting the persistent difficulties in delimiting natural groups within it [2,3]. This instability is largely associated with the historically broad circumscription of *Vernonia* Schreb. [4], which once included nearly 1200 species distributed across both hemispheres [5].

Subsequent studies have consistently demonstrated that *Vernonia sensu lato* is polyphyletic, resulting in a substantially narrowed circumscription of the genus to ca. 20 American species [1,2,6,7]. Although many species formerly placed in *Vernonia* have been transferred to other genera, this process remains incomplete, with approximately 300 species still lacking definitive generic placement [8,9,10]. Of these, 44 species are endemic to Madagascar, and recent molecular evidence indicates that they represent multiple distinct lineages rather than a monophyletic group [11].

Micromorphological traits (e.g., trichomes, anther appendages, cypselae, and pollen morphology) have been used extensively in the classification and taxonomy of Vernonieae, including Malagasy *Vernonia* [12,13,14,15,16,17,18,19,20,21,22,23]. The pollen morphology of this tribe has been extensively documented using traditional approaches based on a limited number of non-standardised characters, primarily emphasising apertures and ornamentation [20,21,22,23]. However, more recent studies have introduced methodological and morphological advances that have expanded the palynological framework applied to taxonomic interpretation [21,22]. Despite the rich diversity of pollen morphology, modern palynological techniques, have yet to be used to study the ultrasculpture and ultrastructure of Malagasy *Vernonia*. This study aims to provide the first comprehensive and standardised palynological assessment of Malagasy *Vernonia* to describe the diversity of pollen among these species, test morphological groupings and pollen types, and explore the taxonomic implications for an ongoing, necessary generic re-circumscription. Focusing on Malagasy taxa is warranted given their high endemism, unique evolutionary history, and unresolved taxonomic status within Vernonieae. A comparison between the standardised palynological assessment used in this study and previous studies is provided in the Discussion.

## 2. Results

### 2.1. General Description of Vernonia from Madagascar

**Figure 1:**
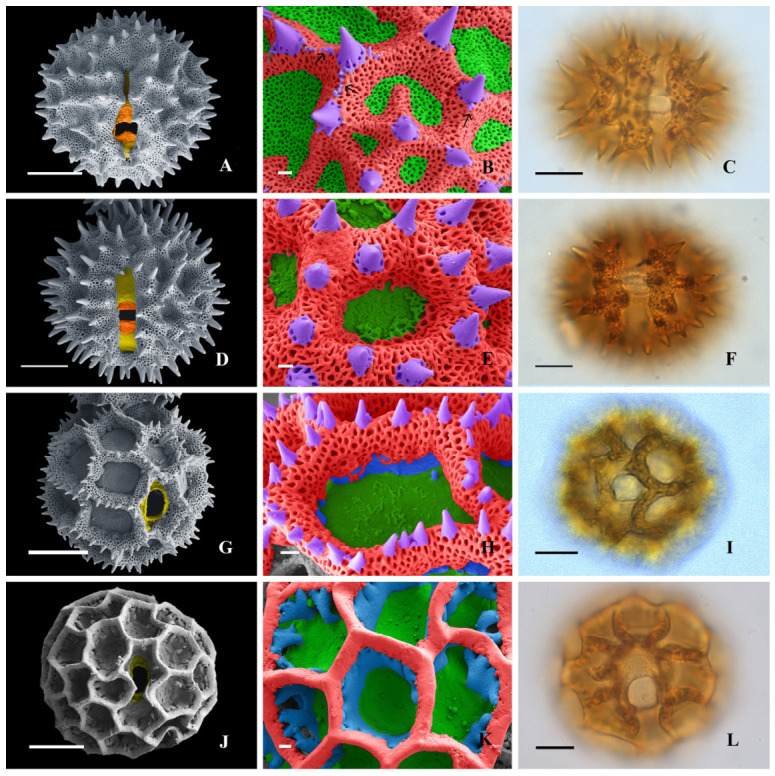
Pollen types of *Vernonia* from Madagascar in equatorial view. (**A**–**C**) Pollen type Ia. (**A**) *Vernonia ikongensis*, SEM general view of the apertural area and mesocolpium. (**B**) *Vernonia isalensis*, SEM detail of the mesocolpium. (**C**) *Vernonia alleizettei*, LM general view of apertural area and mesocolpium. (**D**–**F**) Pollen type Ib. (**D**) *Vernonia seyrigii*, SEM general view of the apertural area. (**E**) *Vernonia sambiranensis*, SEM detail of the mesocolpium. (**F**) *Vernonia seyrigii*, LM general view of apertural area and mesocolpium. (**G**–**I**) Pollen type IIb, *Vernonia neoperrieriana*. (**G**) SEM general view of the mesocolpium and apertural area. (**H**) SEM detail of the mesocolpium. (**I**) LM general view of apertural area and mesocolpium. (**J**–**L**) Pollen type IIa. (**J**) *Vernonia bojeri*, SEM general view of the mesocolpium and apertural area. (**K**,**L**) *Vernonia platylepis*, (**K**) SEM detail of the mesocolpium; (**L**) LM general view of apertural area and mesocolpium, equatorial view. Yellow = exoaperture, orange = mesoaperture, green = depression (sublacunae/lacunae), red = ridges (lophae), purple = spines, black arrow = nanospines, blue = columellae. Scale bar—10 μm (**A**,**C**,**D**,**F**,**G**,**I**,**J**,**L**), 1 μm (**B**,**E**,**H**,**K**).

**Figure 2:**
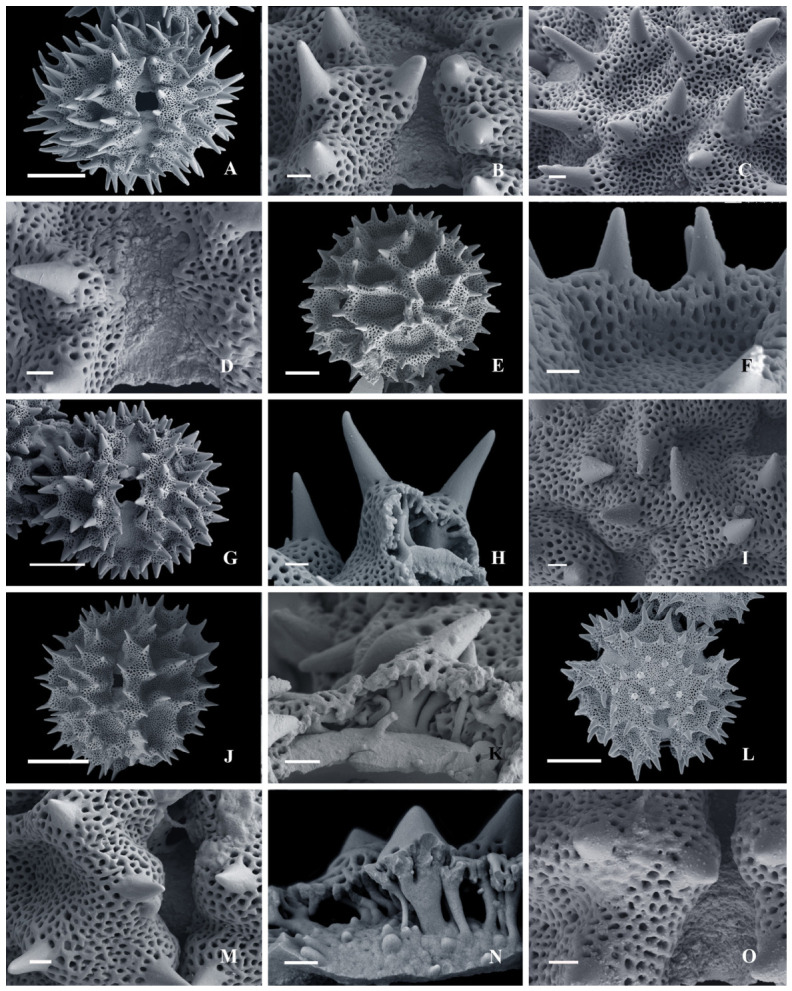
SEM of pollen type Ia of *Vernonia* from Madagascar. (**A**–**C**) *Vernonia alleizettei*. (**A**) General view, equatorial view. (**B**) Detail of the apertural area, equatorial view. (**C**) Detail of the apocolpium, polar view. (**D**) *Vernonia ampandrandavensis*, detail of the apertural area, equatorial view. (**E**,**F**) *Vernonia betonicifolia*. (**E**) General view of the mesocolpium, equatorial view. (**F**) Detail of the ultrasculpture, polar area. (**G**,**H**) *Vernonia betsimisaraka*. (**G**) General view, equatorial view. (**H**) Fractured pollen grain. (**I**) *Vernonia carnotiana*, detail of the apocolpium, polar view. (**J**) *Vernonia decaryana*, general view, equatorial view. (**K**,**L**) *Vernonia diversifolia*. (**K**) Fractured pollen grain. (**L**) General view, polar view. (**M**) *Vernonia homolleae*, detail of the apertural area, equatorial view. (**N**) *Vernonia humillima*, fractured pollen grain. (**O**) *Vernonia ikongensis*, detail of the apertural area, equatorial view. Scale bars—10 μm (**A**,**G**,**J**,**L**), 5 μm (**E**), 1 μm (**B**–**D**,**F**,**H**,**I**,**K**,**M**–**O**).

**Figure 3:**
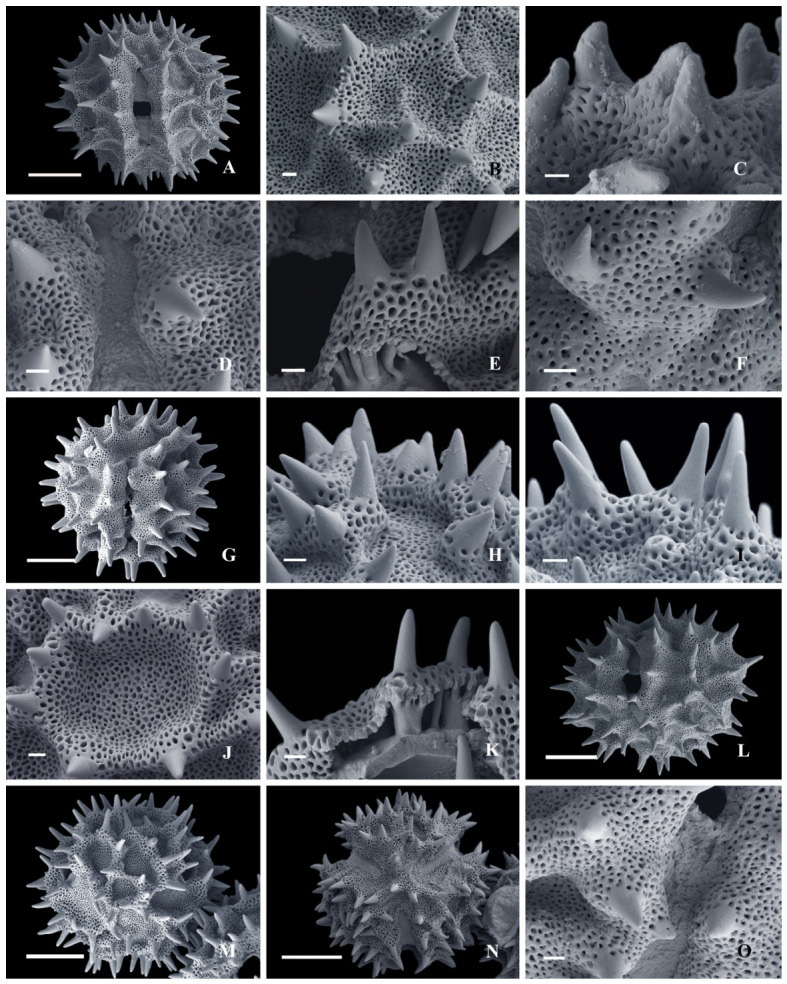
SEM of pollen type Ia of *Vernonia* from Madagascar. (**A**,**B**) *Vernonia isalensis*. (**A**) General view, equatorial view. (**B**) Detail of the apocolpium, polar view. (**C**) *Vernonia leandrii*, detail of the ultrasculpture, polar area. (**D**,**E**) *Vernonia lemurica*. (**D**) Detail of the apertural area, equatorial view. (**E**) Fractured pollen grain. (**F**) *Vernonia manongarivensis*, detail of the apocolpium, polar view. (**G**) *Vernonia monantha*, general view, equatorial view. (**H**) *Vernonia neocoursiana*, detail of the polar area, equatorial view. (**I**) *Vernonia pachyclada*, detail of the ultrasculpture, polar area. (**J**,**K**) *Vernonia pellegrini*. (**J**) Detail of the ultrasculpture, mesocolpium. (**K**) Fractured pollen grain. (**L**) *Vernonia pseudoappendiculata*, general view, equatorial view. (**M**) *Vernonia sakalava*, general view, equatorial view. (**N**) *Vernonia speiracephala*, general view, polar view. (**O**) *Vernonia tanalensis*, Detail of the apertural area, equatorial view. Scale bars—10 μm (**A**,**G**,**L**–**N**), 1 μm (**B**–**F**,**H**–**K**,**O**).

**Figure 4:**
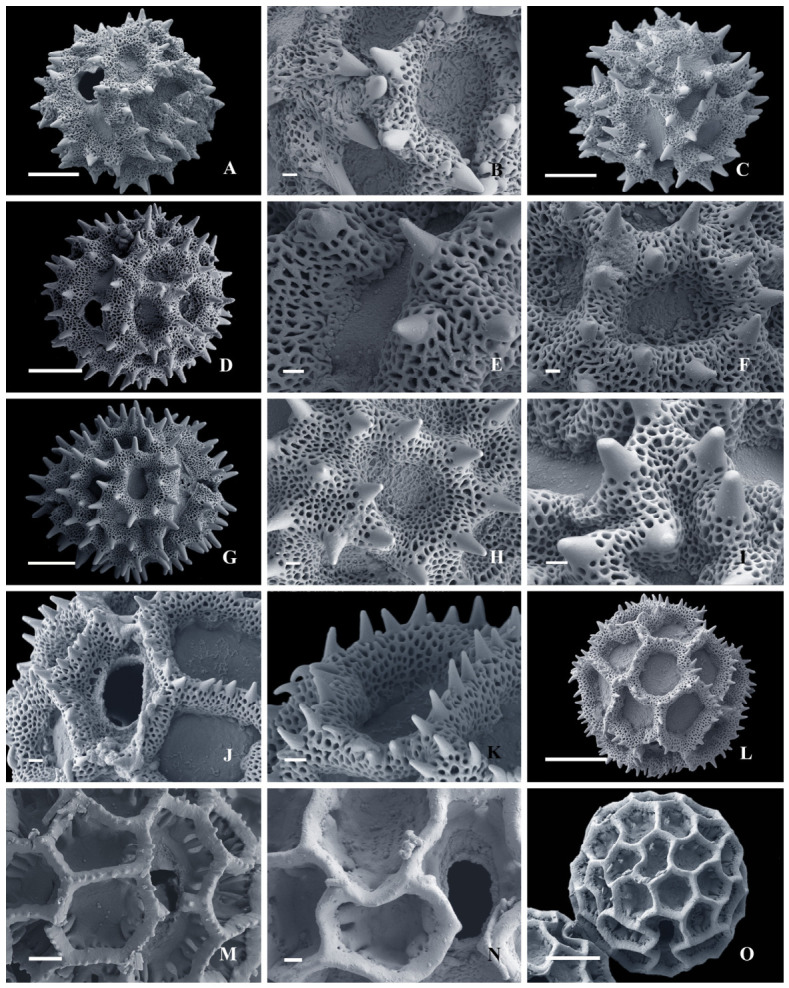
SEM of pollen type Ib (**A**–**I**), IIa (**I**–**L**), IIb (**M**–**O**) of *Vernonia* from Madagascar. (**A**–**C**) *Vernonia mandrarensis*. (**A**) General view of the apertural area and mesocolpium, equatorial view. (**B**) Detail of the ultrasculpture, mesocolpium. (**C**) General view of the apocolpium, polar view. (**D**–**F**) *Vernonia sambiranensis*. (**D**) General view of the mesocolpium and apertural area, equatorial view. (**E**) Detail of the apertural area, equatorial view. (**F**) Detail of the ultrasculpture, mesocolpium. (**G**–**I**) *Vernonia seyrigii*. **G**. General view of the mesocolpium and apertural area, equatorial view. (**H**) Detail of the ultrasculpture, mesocolpium. (**I**) Detail of the apocolpium, polar view. (**J**–**L**) *Vernonia neoperrieriana*. (**J**) Detail of the apertural area and mesocolpium, equatorial view. (**K**) Detail of the ultrasculpture, mesocolpium. (**L**) General view of the apocolpium, polar view. (**M**) *Vernonia ambrensis*, detail of the mesocolpium and apertural area, equatorial view. (**N**,**O**) *Vernonia bojeri*, detail of the mesocolpium and apertural area, equatorial view. (**O**) General view of the apocolpium, polar view. Scale bars—10 μm (**A**,**C**,**D**,**G**,**L**,**O**), 5 μm (**M**), 1 μm (**B**,**E**,**F**,**H**–**K**,**N**).

**Figure 5:**
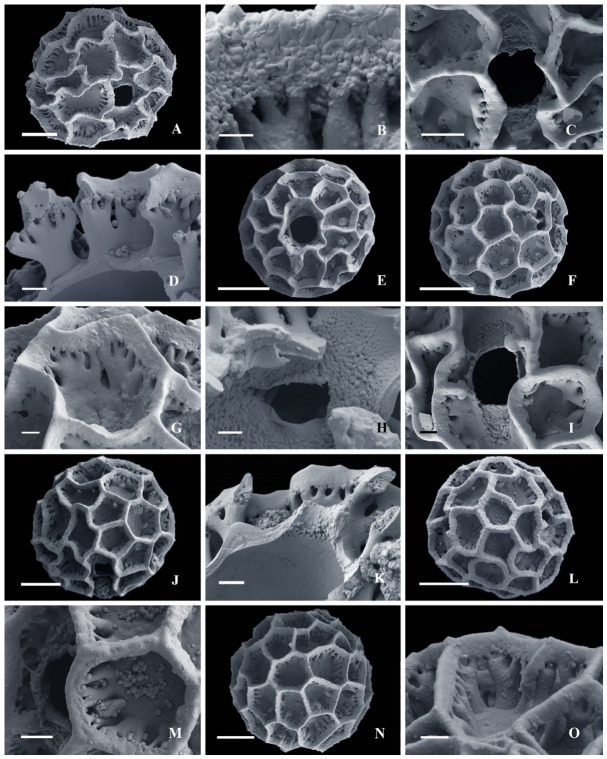
SEM of pollen type IIb of *Vernonia* from Madagascar. (**A**,**B**) *Vernonia cephalophora*. (**A**) General view, equatorial view. (**B**) Detail of the ultrasculpture. (**C**,**D**) *Vernonia kenteocephala*. (**C**) Detail of the apertural area, equatorial view. (**D**) Fractured pollen grain. (**E**,**F**) *Vernonia latisquamata*. (**E**) General view, equatorial view. (**F**) General view, equatorial view. (**G**,**H**) *Vernonia mecistophylla*. (**G**) Detail of the ultrasculpture, mesocolpium. (**H**) Fractured pollen grain. (**I**–**K**) *Vernonia platylepis*. (**I**) Detail of the apertural area, equatorial view. (**J**) General view of the apocolpium, polar view. (**K**) Fractured pollen grain. (**L**,**M**) *Vernonia tropophila*. (**L**) General view, equatorial view. (**M**) Detail of the apertural area, equatorial view. (**N**,**O**) *Vernonia vohemarensis*. (**N**) General view, polar view. (**O**) Detail of the ultrasculpture, mesocolpium. Scale bars—20 μm (**E**,**F**), 10 (**A**,**J**,**L**,**N**), 10 μm (**C**), 2 μm (**D**,**G**–**I**,**K**,**M**,**O**), 1 μm (**B**).

Pollen grains are monads, of medium to large size, measuring 35.1 (38.1 ± 1.5) 41.0 × 32.7 (35.5 ± 1.4) 38.2 × 34.0 (37.1 ± 1.5) 40.2 µm (Supplementary Material Table S1), radially symmetrical, isopolar, oblate spheroidal, amb subtriangular to triangular, equatorial outline circular to ellipsoidal, angulaperturate, polar area very large to very small. Apertures 3-zonocolporate, longicolporate (Figure 1A,D, Figure 2A,G,J and Figure 3L) to brevicolporate (Figure 1G,J, Figure 4J,N and Figure 5I), apices pointed or rounded, colporus margo perforate, colporus membrane granulate, protruding aperture absent, operculum not observed; apertural lacunae present, very small to medium (Supplementary Material Table S2), lacunae constriction present or absent, interlacunar ridges present or absent; endoaperture present, class of endoaperture lalongate, circular, lalongate to very lalongate, endoaperture terminals acute or rounded. Exine thick 4.6 (7.5 ± 2.3) 10.3 µm, sexine thicker than nexine. Sculpture sublophate (subechinolophate, with lacunae or sublacunae) or lophate (echinolophate to psilolophate). Sublophae irregular to polygonal, nano- to microreticulate, muri perforate or non-perforate; spine present, base non-constricted, apices acute to rounded, nanospines present or absent; or lophae psilolophate to nano- to microrugulate, nano- to microechinate, perforation and granules present or absent. Tectum semitectate to tectate-perforate, supratectum elements present (spines, microspines, nanospines) or absent, infratectum columellate, columellae simple, thin to digitate thick (Figure 2H,K,N, Figure 3E,K and Figure 5D,K), incomplete columellae and granules present (Figure 2K,N and Figure 3K); foot layers continuous to discontinuous, compact to granulate; endexine thick to thin. Digitate columella supporting the spines in a concordant pattern and simple columellae supporting the pluricolumellate lophae (Figure 2H,K,N and Figure 3E,K) or thick to thin digitate to simple columellae supporting the lophae (Figure 5B,D,G,K).

A summary of the measurements is shown in Supplementary Materials Tables S1 and S2, and additional observations and descriptions are provided in Supplementary Materials Tables S3 and S4. Additionally, an identification key for pollen types and subtypes is presented in the Section 2 to facilitate interpretation and reproducibility.

### 2.2. Pollen Grain Sizes, Apertures, Exine and Pollen Types

#### 2.2.1. Type I

Subechinolophate—pollen grains medium-sized, 32.2 (40.6 ± 4.2) 48.8 × 28.9 (36.9 ± 4.00) 44.8 × 31.1 (39.9 ± 4.4) 48.5 µm (Supplementary Material Table S1), polar area medium-sized, small or very small; apertures 3-longicolporate (Figure 1A,D and Figure 2A,G,J), colpori length medium, long to very long, 21.5 (25.7 ± 2.1) 29.9 µm (Supplementary Material Table S2); mesoaperture present (Figure 1A,D, Figure 2D and Figure 3D); endoaperture terminals rounded to acute; sculpture subechinolophate (Figure 1B, Figure 2C,E,I and Figure 3B,H,J); columellae non-visible; sculpture subechinolophate, exine thin 4.6 (6.2 ± 0.7) 7.6 µm, sexine thin, 2.9 (4.1 ± 0.5) 5.2 µm, index sexine/nexine, 1.3 (2.0 ± 0.3) 2.7 µm; tectum tectate-perforate (Figure 1A–F, Figure 2, Figure 3 and Figure 4A–I, Supplementary Materials Tables S1–S3).

##### Subtype Ia

Subechinolophate with sublacunae—polar area medium-sized, small or very small; colporus medium-sized, large to very large, length 21.6 (25.3 ± 1.8) 29.0 × width 3.6 (5.3 ± 0.8) 7.2 µm, apices pointed or rounded; endoaperture width 5.6 (7.7 ± 1.0) 9.7 × length 2.9 (4.8 ± 0.9) 6.6 µm, terminals rounded to acute; sublacunae nano- to microreticulate, nano- to microreticulate-perforate or perforate, nanoreticulum complete, muri of the reticulum straight; exine 4.1 (6.1 ± 0.6) 7.4, tectum tectate-perforate. (Figure 1A–C, Figure 2 and Figure 3, Supplementary Materials Tables S1–S3).

Species: *Vernonia alleizettei* Humbert, *V. ampandrandavensis* Humbert, *V. andapensis* Humbert, *V. betonicifolia* Baker, *V. betsimisaraka* Humbert, *V. carnotiana* Humbert, *V. decaryana* Humbert, *V. diversifolia* Bojer ex DC. subsp. *diversifolia*, *V. diversifolia* subsp. *ikopae* (Drake) Humbert, *V. homolleae* Humbert, *V. humillima* Humbert, *V. ikongensis* Humbert, *V. isalensis* Humbert, *V. leandrii* Humbert, *V. lemurica* Humbert, *V. manongarivensis* Humbert, *V. monantha* Humbert, *V. neocoursiana* Humbert, *V. pachyclada* Baker, *V. pellegrinii* Humbert, *V. pseudoappendiculata* Humbert, *V. sakalava* Humbert, *V. speiracephala* Baker, and *V. tanalensis* Baker.

Polar area medium-sized, small or very small. Colporus medium-sized (Supplementary Material Table S2), large or very large, non-constricted, apices acute or rounded. Endoaperture circular to lalongate, lalongate to very lalongate or very lalongate (Supplementary Material Table S2), terminals acute. Lophae nanoreticulate, nanoreticulate to microreticulate, nanoreticulate-perforate, nanoreticulate to microreticulate-perforate, nano-spines present (Figure 1B) or absent, spine apices acute to rounded; sublacunae irregular or polygonal, perforate (Figure 3G), nanoreticulate (Figure 2E,F), nanoreticulate-perforate (Figure 2L and Figure 3B,J,M), nanoreticulate to microreticulate (Figure 2C) or nanoreticulate to microreticulate-perforate (Figure 2J), tectum tectate-perforate.

##### Subtype Ib

Subechinolophate with lacunae—polar area very small-sized; colporus very large, length 28.0 (29.2 ± 0.2) 30.4 × width 6.1 (6.6 ± 0.2) 7.1 µm, apices rounded; endoaperture width 8.4 (9.0 ± 0.2) 9.5 µm × length 5.8 (6.2 ± 0.1) 6.6 µm, terminals rounded; lacunae granulate-perforate, nanoreticulum incomplete, muri of the reticulum curved; exine 6.7 (7.1 ± 0.2) 7.5 µm, tecum tectate-perforate (lophae) to semitectate (lacunae). (Figure 1D–F and Figure 4A–I, Supplementary Materials Tables S1–S3).

Species: *Vernonia mandrarensis* Humbert, *V. sambiranensis* Humbert, and *V. seyrigii* Humbert.

Pollen grains with irregular or polygonal lacunae and spine apices acute or rounded.

#### 2.2.2. Type II

Lophate—pollen grains medium to large sized 41.9 (55.9 ± 7.1) 69.8 × 39.4 (53.9 ± 7.3) 68.3 × 41.2 (55.3 ± 7.0) 69.1 µm, polar area large-sized to very large; apertures 3-brevicolporate, colpori length small to very small 9.2 (10.6 ± 0.6) 11.9 µm; mesoaperture mostly present; endoaperture terminals rounded; sculpture lophate (psilolophate to echinolophate); columellae visible, rarely non-visible; exine thick 8.6 (11.2 ± 1.2) 13.7 µm, sexine thick 6.5 (8.8 ± 1.1) 13.7 µm, index sexine/nexine 2.8 (3.8 ± 0.5) 4.8 µm; tectum semitectate to tectate perforate (Figure 1G–L, Figure 4J–O and Figure 5, Supplementary Material Tables S1, S2 and S4).

##### Subtype IIa

Psilolophate to nano- to microechinolophate—lophae perforate-granulate, nano- to microrugulate-granulate or nano- to microechinate with sparse perforation and granules, colporus with rounded apices, mesoaperture present, tectum semitectate, columellae visible. (Figure 1J–L, Figure 4M–O and Figure 5, Supplementary Materials Tables S1, S2 and S4)

Species: *Vernonia ambrensis* Humbert, *V. bojeri* Less., *V. cephalophora* Humbert, *V. kenteocephala* Baker, *V. latisquamata* (Humbert) Humbert, *V. mecistophylla* Baker, *V. platylepis* Drake, *V. tropophila* Humbert, and *V. vohemarensis* Humbert.

Pollen grains are medium to large-sized, amb circular to subtriangular, polar area large to very large. Apertural lacunae very small (Figure 5E), small (Figure 4M,N) or medium (Figure 5C,I), lacunae constriction present (Figure 4M,N) or absent (Figure 5E), interlacunar ridges present (Figure 4M and Figure 5E) or absent (Figure 5A). Colporus small (Figure 4M,N) or very small (Figure 5E), apices rounded or slightly acute. Endoaperture lolongate, circular, lalongate to very lalongate (Supplementary Material Table S2), endoaperture terminals rounded. Ornamentation echinolophate (nano- to microechinate) (Figure 4M) with sparse perforation and granules (Figure 5M), psilolophate (Figure 5E,I), nano- to microrugulate-granulate present (Figure 5A,B) or absent; lacunae smooth or granulate. Columellae simple to digitate (Figure 4M and Figure 5B) or digitate (Figure 5D,G,O).

##### Subtype IIb

Echinolophate—Lophae nano- to microreticulate, colporus with acute apices, mesoaperture absent, tectum semitectate to tectate perforate, columellae non-visible—Species: *Vernonia neoperrieriana* Humbert. (Figure 1G–I and Figure 4J–L, Supplementary Materials Tables S1, S2 and S4).

Pollen grains are medium-sized, polar area large, apertural lacunae very small, lacunae constriction absent, interlacunar ridges absent. Colporus very small, endoaperture circular to lalongate. Spines 1.5 μm, lacunae granulate-nanorugulate (Figure 1G–I and Figure 4J–L, Supplementary Materials Tables S1, S2 and S4).

### 2.3. Identification Key for the Types and Subtypes

1. Pollen grains medium-sized [i.e., 32.2 (40.6 ± 4.2) 48.8 × 28.9 (36.9 ± 4.00) 44.8 × 31.1 (39.9 ± 4.4) 48.5 µm], polar area medium-sized, small or very small; apertures 3-longicolporate, colporus length medium, long to very long [i.e., 21.5 (25.7 ± 2.1) 29.9 µm]; mesoaperture present; endoaperture terminals rounded to acute; sculpture subechinolophate; columellae inconspicuous; exine thin [i.e., 4.6 (6.2 ± 0.7) 7.6 µm], sexine thin [i.e., 2.9 (4.1 ± 0.5) 5.2 µm], sexine/nexine index 1.3 (2.0 ± 0.3) 2.7 µm; tectum tectate-perforate 2 (Type I –Subechinolophate)

1′. Pollen grains medium- to large-sized [i.e., 41.9 (55.9 ± 7.1) 69.8 × 39.4 (53.9 ± 7.3) 68.3 × 41.2 (55.3 ± 7.0) 69.1 µm], polar area large-sized to very large; apertures 3-brevicolporate, colporus length small to very small [i.e., 9.2 (10.6 ± 0.6) 11.9 µm]; mesoaperture mostly present; endoaperture terminals rounded; sculpture psilolophate to echinolophate; columellae conspicuous, rarely inconspicuous; exine thick [i.e., 8.6 (11.2 ± 1.2) 13.7 µm], sexine thick [i.e., 6.5 (8.8 ± 1.1) 13.7 µm], sexine/nexine index 2.8 (3.8 ± 0.5) 4.8 µm; tectum semitectate to tectate-perforate 3 (Type II—Lophate)

2. Sculpture with sublacunae, polar area medium-, small- or very small-sized; colporus medium-, large- to very large-sized [i.e., length 21.6 (25.3 ± 1.8) 29.0 × width 3.6 (5.3 ± 0.8) 7.2 µm], apices acute or rounded; endoaperture width 5.6 (7.7 ± 1.0) 9.7 × length 2.9 (4.8 ± 0.9) 6.6 µm, terminals acute to rounded; sublacunae nano- to microreticulate, nano- to microreticulate-perforate or perforate, nanoreticulum complete, muri of the reticulum straight; exine 4.1 (6.1 ± 0.6) 7.4 µm Type Ia—Subechinolophate with sublacunae

2′. Sculpture with lacunae, polar area very small-sized; colporus very large-sized [i.e., length 28.0 (29.2 ± 0.2) 30.4 × width 6.1 (6.6 ± 0.2) 7.1 µm], apices rounded; endoaperture width 8.4 (9.0 ± 0.2) 9.5 × length 5.8 (6.2 ± 0.1) 6.6 µm, terminals rounded; lacunae granulate-perforate, nanoreticulum incomplete, muri of the reticulum curved; exine 6.7 (7.1 ± 0.2) 7.5 µm Ib—Subechinolophate with lacunae

3. Sculpture psilolophate to nano- to microechinolophate, lophae perforate-granulate, nano- to microrugulate-granulate or nano- to microechinate with sparse perforation and granules, colporus with rounded apices, mesoaperture present, tectum semitectate, columellae conspicuous Pollen Type IIa—Psilolophate to nano- to microechinolophate

3′. Sculpture echinolophate, lophae nano- to microreticulate, colporus with acute apices, mesoaperture absent, tectum semitectate to tectate perforate, columellae inconspicuous Type IIb—Echinolophate

### 2.4. Principal Component Analysis (PCA)

**Figure 6:**
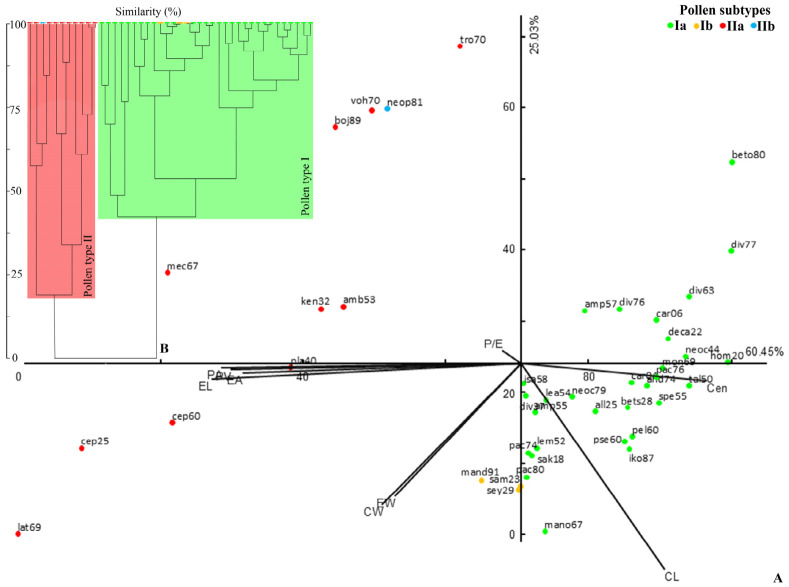
Multivariate analysis. (**A**) Principal component analysis biplot of the pollen grain metric variables and classes/indices of Malagasy *Vernonia* specimens. (**B**) Dendrogram built from the cluster analysis (Euclidean distance), similarity information remaining (%).

PCA suggests the separation of the species into four groups, based on seven metric variables and two classes/indices (Figure 6A); correlation coefficients are shown in Supplementary Material Table S4. The first two axes accounted for 85.48% of the total variability of the quantitative data analysed. The first axis explained 60.45% of the variance and was mainly associated with EA, PA, PV, EL and Cen (see Section 4 and Section 4.3). The second axis summarised 25.03% of the total variability, with P/E, CL, CW and EW being the variables that contributed most to this axis.

*Vernonia cephalophora* (cep25, cep60), *V. latisquamata* (lat69), *V. platylepis* (pla40) and *V. mandrarensis* (mand91), *V. sambiranensis* (sam23), *V. seyrigii* (sey29), represent the highest values relative to endoaperture dimensions and colporus width was located on the bottom left side of the PCA associated with high values of EA, PA, CW, EL, EW. *Vernonia ambrensis* (amb53), *V. kenteocephala* (ken32), *V. mecistophylla* (mec67), *V. bojeri* (boj89), *V. neoperrieriana* (neop81), *V. tropophila* (tro70), *V. vohemarensis* (voh70) showed the smallest colporus length (CL) were located on the upper left side of the PCA.

Due to their high Cen and CL values, *Vernonia pachyclada* (pac80, pac74, pac76), *V. diversifolia* (div37), *V. isalensis* (isa58), *V. manongarivensis* (mano67), *V. sakalava* (sak18), *V. lemurica* (lem52), *V. ikongensis* (iko87), *V. pellegrinii* (pel60), *V. pseudoappendiculata* (pse60), *V. alleizettei* (all25), *V. ampandrandavensis* (amp55), *V. andapensis* (and74), *V. betsimisaraka* (bets28), *V. leandrii* (lea54), *V. neocoursiana* (neoc79), *V. speiracephala* (spe55) and *V. tanalensis* (tan50) placed on the bottom right side of the PCA with lalongate to very lalongate endoaperture. Specimens with the smallest pollen grains were located mainly on the top right side of the graph. These specimens also had the lowest EA, PA, PV, CW, EW, EL and EL values: *V. betonicifolia* (beto80), *V. diversifolia* (div77, div 76, div63), *V. ampandrandavensis* (amp57), *V. carnotiana* (car06), *V. decaryana* (dec22), *V. homolleae* (hom20), *V. neocoursiana* (neo44).

### 2.5. Cluster Analysis (UPGMA and Euclidean Distance)

We identified two groups of specimens with 0% similarity (Figure 6B, Supplementary Material Table S5) on the cluster analysis. One group had a similarity greater than 20%. It comprised the species with the largest pollen grains, highest endoaperture dimensions and the smallest colporus length (*Vernonia ambrensis* [amb53], *V. bojeri* [boj89], *V. cephalophora* [cep25, cep60], *V. kenteocephala* [ken32], *V. latisquamata* [lat69], *V. mecistophylla* [mec67], *V. neoperrieriana* [neop81], *V. platylepis* [pla40], *V. tropophila* [tro70], *V. vohemarensis* [voh70]). The other group had a similarity greater than 45%, and the remaining species with the smallest pollen grains, largest colporus and the endoaperture values and the highest endoaperture class (*V. alleizettei* [all25], *V. ampandrandavensis* [amp55, amp57], *V. andapensis* [and74], *V. betonicifolia* [bet80], *V. betsimisaraka* [bets28], *V. carnotiana* [car06], *V. decaryana* [dec22], *V. diversifolia* [div37, div77, div 76, div63], *V. ikongensis* [iko87], *V. isalensis* [isa58], *V. homolleae* [hom20], *V. leandrii* [lea54], *V. lemurica* [lem52], *V. manongarivensis* [mano67], *V. neocoursiana* [neo44], *V. pachyclada* [ac80, pac74, pac76], *V. pellegrinii* [pel60], *V. pseudoappendiculata* [pse60], *V. sambiranensis* [sam23], *V. sakalava* [sak18], *V. seyrigii* [sey29], *V. speiracephala* [spe55], *V. neocoursiana* [neoc79], and *V. tanalensis* [tal50]).

## 3. Discussion

### 3.1. Pollen Morphology of Malagasy Vernonia

To define the pollen types in this study, we used a combination of 18 characters for both types (i.e., pollen grain size, axis measurements, polar area size, colporus class, size and measurements, endoaperture terminals, exine ornamentation, columellae appearance, exine thickness, sexine thickness, index sexine/nexine, and tectum type). We also present for the first time a morphometric analysis (i.e., of the axis, apertures, and exine layers) and endoaperture descriptions to assist in the delimitation of the pollen types. These pollen types were also corroborated by the multivariate statistical analysis (Figure 6). The PCA results indicate that the first axis is primarily associated with size-related variables (e.g., pollen grain dimensions and aperture measurements), while the second axis reflects variation in apertural and exine-related characters, suggesting that both quantitative size parameters and qualitative morphological traits contribute to species differentiation. The representatives of pollen type I present a similarity greater than 45% (Figure 6B), and the pollen subtype Ia is located on the right side of the plot. In contrast, pollen type Ib is grouped on the bottom left side of the graph (Figure 6A) with a similarity close to 90% (Figure 6B). The representatives of pollen type II (subtypes a and b) are placed on the left side of the graph (Figure 6A) and present a similarity greater than 20%.

The last palynological analysis that included Malagasy representatives of *Vernonia* was conducted more than 45 years ago [17,23]. Jones [17] described five species also analysed in the present study (*Vernonia bojeri*, *V. diversifolia*, *V. homolleae*, *V. kenteocephala*, and *V. mecistophylla*) plus three other species currently recognised in other genera [*Bechium nudicaule* (Less.) H.Rob., *Bothriocline madagascariensis* (DC.) C.Jeffrey (pollen descriptions available [22]) and *Bechium rhodolepsis* (Baker) H.Rob.]. He used 12 non-standardised characters (ornamentation, sculpture, ultra-sculpture, aperture, tectum, columellae, and spines, presence/absence) and grouped the species only by pollen type, without any statistical multivariate analyses [17]. We have observed some terminological inconsistencies in the selected pollen characters (e.g., echinolophate is a type of lophate sculpture, and “micropunctate and semitectate” is a type of discontinuous tectum) and missing data for some characters (e.g., endoaperture type and apertural lacunae).

The Malagasy taxa were included in two of these pollen types *sensu* Jones [17]: Pollen Type A—echinate to subechinolophate, tricolporate with a continuous, micropunctate tectum, spines on the ridges or muri of the subechinolophate grains (*V. diversifolia*, and *V. homolleae*); and Pollen Type E—lophate or subechinolophate, semi-tectate with elevated geometrically arranged muri, supported by conspicuous columellae, lacunae regularly spaced, germinal pores without distinguishing features (*V. bojeri*, *V. kenteocephala*, and *V. mecistophylla*) [17].

The pollen types identified in the present study do not have any comparative basis to those classified by Jones [17]. Notably, the pollen types *sensu* Jones [17] have some overlap with ours (i.e., subechinolophate); however, some characters we have documented in the present study were not considered in the classification proposed by Jones (i.e., number of apertures, presence/absence of spines, and appearance of the columella). Overlap in the pollen type characters has been reported previously as a problem for the palynotaxonomy of Vernonieae [5]. Among these, pollen type E poses a challenge because it includes pollen grains with lophate or subechinolophate sculpture, which is incompatible with the type classification provided in our study (e.g., Type I—Subechinolophate, and Type II—Lophate; [17]). Such overlap and terminological inconsistencies prevent us from comparing our morphological and morphometric data with the previous analysis performed by Jones [17] and renders the two classification systems incompatible. We revisited the pollen types sensu Jones [17] and we provide a revision of the characters and the character states, which were updated according to the terminology used in the present study (see Section 4 and Section 4.2) to present the terminological inconsistencies, overlaps and missing data problems (see Supplementary Material Table S6).

Kingham [23] have also analysed *Vernonia bojeri* and *V. diversifolia* plus seven other species currently recognised in other genera (*Bechium nudicaule*, *Distephanus glutinosus* (DC.) H.Rob. & B.Kahn, *D. nummulariifolius* (Klatt) H.Rob. & B.Kahn, *D. subluteus* (Scott Elliot) H.Rob. & B.Kahn, *D. trinervis* Bojer ex DC., *Gymnanthemum appendiculatum* (Less.) H.Rob. and *Linzia* glabra Steetz). Both *Vernonia* species are included in the pollen type VI sensu Kinghan [23] (pollen grains subechinolophate, tending to echinate, tricolporate, micropuncta present). These results corroborate the description of *V. diversifolia* into the pollen type I (sublophate) in the present study. However, the *V. bojeri* specimen analysed here is included in the pollen type II (lophate).

### 3.2. Apertural System and Exine Structure

The pollen grains of the species of *Vernonia* occurring in Madagascar are characterised by 3-colporate apertures, mesoaperturate (mostly present), thick exine, sexine thicker than the nexine, and subechinolophate or lophate sculpture. Previous observations in *Vernonia* (including non-Malagasy representatives) have not reported the presence of a mesoaperture. This pollen morphological character has been investigated in other evolutionary studies of Compositae pollen [24]. This structure is here described for the first time in the context of a comprehensive palynological classification for species of *Vernonia*. This compound aperture is characterised by the presence of a middle section aperture in which there are both an exoaperture and an endoaperture present [25]. Based on the characteristic Compositae exine ultrasculpture, the mesoapertural system involves a complex stratification of the foot layer, plus the outer layer of the endexine. At the same time, the exoaperture (i.e., colpus, porus) is comprised of the tectum and infratectum structures, and the endoaperture, the inner layer of the endexine [26,27].

Mesoapertures were found in the majority of the Malagasy species of *Vernonia* described here, except in Pollen Type IIb (*V. neoperrieriana*). Additionally, the mesoaperture has been described as ‘sometimes present’ in Vernonieae [25] and seems to be more widely represented in the tribe. Comprehensive studies are needed to investigate the presence of the mesoapertural system in other taxa and geographic regions. Tormo-Molina and Ubera-Jimenez [27] noted a knowledge gap regarding the pollen apertural system in Compositae.

To date, the mesoapertural system has not been comprehensively described or characterised within Compositae [25]. Its taxonomic and evolutionary significance has mainly been discussed in terms of its presence across various tribes and clades (i.e., Barnadesieae, Calenduleae, Cardueae, Cichorieae, Dicomeae, Gundelieae, Hecastocleideae, Mutisieae, Oldenburgieae, Stifftia clade, Tarchonantheae, and Vernonieae) [25]. However, the specific ecological function of the mesoaperture remains unknown. It is important to note that structures related to pollen apertures in general have been associated with roles in pollination, such as pollen-stigma recognition [28,29], and in providing resistance to environmental constraints, for example, through maintaining water balance and the harmomegathic function [29,30]. However, whether the mesoaperture itself fulfils similar functions, or has distinct taxonomic or evolutionary significance within Vernonieae and Compositae, remains to be determined. Further research will be necessary to clarify the functional, taxonomic, and evolutionary implications of this structure.

### 3.3. Taxonomic History of Vernonia from Madagascar and Palynological Implications

The only modern taxonomic treatment proposed for the Malagasy Vernonieae was published in the last century by Humbert [12] in *Flores de Madagascar et des Comores*. He analysed 96 species, circumscribed into six morphological groups based on their habits, phyllaries, and floret colours. Almost 25% of these species (23 species, including the whole morphological group 5 and 6 *sensu* Humbert [12]) have now been re-circumscribed into *Distephanus* Cass and another 25% of the species in other genera (*Gymnanthemum* Cass.—14, *Bechium* DC.—3, *Cyanthillium* Blume—1). With the exception of *Distephanus* (groups 5 and 6), which was not part of the present study, the pollen types delimited here are not consistent with groups defined by Humbert [12], since at least one species from each of Humbert’s groups is represented in pollen type I or II defined in this study.

Like all species of *Vernonia* from the Eastern Hemisphere, the Malagasy taxa are still underrepresented in phylogenetic studies. The first molecular phylogenetic study, with a focus on that region, was recently published using one nuclear locus (nrITS) and three plastid loci (*ndh*F, *psb*A-*trn*H, and *trn*L), focusing on *Distephanus* (Distephaneae, sister of the clade Vernonieae + Moquinieae) [11]. These data confirm the polyphyly of *Vernonia* and the non-monophyly of the Malagasy *Vernonia* [11].

The current molecular data available for the Malagasy representatives include eight species, five of which have their pollen described here (*V. alleizettei*, *V. neoperrieriana*, *V. seyrigii*, *V. leandrii*, and *V. latisquamata*), and another three are presented only as *Vernonia* sp. [11]. Most of these taxa are pollen type I representatives, and only one (*V. neoperrieriana*) is representative of pollen type II. Since the species of *Vernonia* from Madagascar are still insufficiently sampled in molecular phylogenetic studies, it is only possible to infer, from these results, that pollen type I occurs in more than one lineage of this group. A more thorough exploration of the evolutionary significance of these pollen characters is not yet possible.

The distribution of pollen types across Malagasy *Vernonia* suggests that these morphological patterns do not fully correspond to currently recognised taxonomic groupings, reinforcing the complexity of the group and its non-monophyletic nature. Nevertheless, these pollen traits provide useful diagnostic characters and may contribute to refining species delimitation and future taxonomic revisions. Although molecular sampling of Malagasy *Vernonia* is still limited, these palynological traits provide a preliminary basis for future integrative systematic analyses, which could allow the identification of palynological synapomorphies for the clades that will be resolved in the ongoing molecular phylogenetic analyses and could support a new generic re-classification. They may also contribute to discussions of character evolution, lineage differentiation, and the taxonomic placement of currently unassigned species, as broader phylogenomic and plastomic datasets will become available.

## 4. Materials and Methods

### 4.1. Pollen Sampling and Laboratorial Methods

For this study, the taxonomic sampling included 37 (out of 44) currently recognised species of *Vernonia* Schreb. (Appendix A) occurring in Madagascar. For the remaining seven species (*V. ambolensis* Humbert, *V. baillonii* Scott Elliot, *V. betsilensis* Drake, *V. delapsa* Baker, *V. hispidula* Drake, *V. marojejyensis* Humbert, and *V. platylepis* Drake), no suitable samples could be obtained.

Unopened mature flower buds were collected from 46 specimens from the K and P herbaria [31]; all specimens examined are listed in the Specimens Investigated section (Appendix A). Samples were labelled with an abbreviation of the specific epithet followed by the last two numbers of the herbarium voucher (see Supplementary Material Tables S1 and S2, Specimen column; Figure 6; and Appendix A, short name).

Pollen grains were treated with the acetolysis method [32], with modifications [33]. Measurements and photomicrographs were performed under a light microscope, Leica LMD7 Microdissection Microscope (Leica Microsystems GmbH, Wetzlar, Germany), and photomicrographs were taken with a video camera, Leica DFC 7000T (Leica Microsystems GmbH, Wetzlar, Germany), supported by LAS X software, at Royal Botanic Gardens, Kew. The permanent slides for LM were deposited in the pollen reference collection of the Bioimaging Lab, Royal Botanic Gardens, Kew, United Kingdom. For scanning electron microscopy (SEM), the same acetolysed pollen grains prepared for the LM were rinsed and placed on a metal stub with carbon cement and sputter-coated with platinum (10 nm) using a Quorum-Q150 TES Series (Quorum Technologies Ltd., Laughton, UK). Samples were imaged under a Hitachi 8230 Scanning Electron Microscope (Hitachi, Tokyo, Japan), with a 5 kV electron beam, at the Bioimaging Lab, Royal Botanic Gardens, Kew.

### 4.2. Measurements and Terminology

Measurements were taken under light microscopy, using a Leica LMD7 Microdissection Microscope (Leica Microsystems GmbH, Wetzlar, Germany), on 25 randomly selected pollen grains from each specimen. The polar and equatorial axes were measured in the equatorial view, and the equatorial axis in the polar view, excluding spines. Ten measurements of the main pollen morphometric parameters were also made for each specimen: length and width of the colporus, length and width of the endoaperture, length and width of the colporus lacunae (apertural lacunae), size of lophae and muri, spine length and the thickness of the nexine and sexine layers (excluding spines) [23]. Exine measurements were made in the mesocolpium region. Image calibration was carried out using Leica Application Suite X (LAS X) software prior to each measurement session. Measurement variability and error were assessed through descriptive and multivariate statistical analyses, as detailed in Section 4.3.

Pollen terminology follows Punt et al. [26] and Halbritter et al. [29]. Pollen shape classes and amb types follow Erdtman [34], polar area and colpus and lacunae length categories follow Mark et al. [35], and specific sculpturing and apertures type to Vernonieae follow Robinson et al. [18]. The endoaperture classes and sexine/nexine thickness index follow Antonio-Domingues et al. [36]. Exine infratectum followed the terminology proposed by Ferguson and Skvarla [37]. Columellae visibility was based on SEM images of non-fractured pollen grains. The CI (minor and major) and Sx are present in descriptions in the following order: axis—EA × PA × PV; apertures—(width × length). The pollen types were initially defined based on quantitative data and multivariate analyses. They were further supported by primary qualitative characters observable under light microscopy, to provide a robust framework for applied palynological studies. Additionally, we provided a discussion regarding the previous pollen types defined previously [13,23].

### 4.3. Statistical Analyses

To describe and characterise the species, the measurements of the polar and equatorial axes were statistically analysed for the arithmetic mean (x), average standard deviation (Sx), sample standard deviation (s), coefficient of variability (V%), and 95% confidence interval (CI) [38,39].

A principal component analysis (PCA) was performed to explore whether pollen grain characters corroborate species and specimen grouping. Seven metric variables (polar axis [PA], equatorial axis [EA], equatorial axis in polar view [PV], colporus length [CL], colporus width [CW], endoaperture length [EL], endoaperture width [EW]) and two classes/index (endoaperture class [Ecl], shape class [P/E = PA/EA]) were analysed for ordination using FITOPAC [40] and PC-ORD version 7 [41]. To understand the distribution of the variation in the pollen characters among species of *Vernonia*, and how they contributed to potentially grouping the species by morphological similarity, a cluster analysis (CA) was performed using the software PC-ORD version 7 [41]. The selection of morphometric variables for PCA and cluster analyses focused primarily on characters that are widely recognised as informative for pollen taxonomy and morphology in Compositae and in other plant groups, and also as potentially informative for Vernonieae.

## 5. Conclusions

This study represents the first nearly comprehensive characterisation of pollen morphology of Malagasy *Vernonia*—including completely new measurements and descriptions for 37 species. Our work has classified the morphological diversity of pollen in Malagasy Vernonieae into two major categories (Type I and Type II), each with a subtype (A and B). Future work might investigate pollen morphological evolution in Vernonieae through ancestral character state reconstruction with a well-sampled and well-resolved phylogeny. Furthermore, additional genera might be characterised in a large synthesis that explores the diversity of pollen morphology using the standardised approach we present here for all genera of Vernonieae in the Eastern Hemisphere, to evaluate the potential of these characters as taxonomically informative for circumscribing subtribes and segregating genera.

## Data Availability

The permanent slides for LM were deposited in the pollen reference collection of the Bioimaging Lab, Royal Botanic Gardens, Kew, United Kingdom (KMIC-slide barcode for each specimen is available on Specimens Investigated, Appendix A. Other data used in this study are available in Supplementary Materials Tables S1–S6 and Figure S1.

## Supplementary Materials

The following supporting information can be downloaded at https://www.mdpi.com/article/10.3390/plants15121927/s1, Table S1: *Vernonia* dimensions (μm) pollen grains in equatorial and polar view using light microscopy; Table S2: Dimensions (μm) pollen grains aperture, spine, reticulum and exine layers in equatorial view using light microscopy; Table S3: Morphology and ultrasculpture of *Vernonia* pollen grains Type I (*Sublophate*) using light and scanning electron microscopy; Table S4: Morphology and ultrasculpture of *Vernonia* pollen grains Type II (*Lophate*) using light and scanning electron microscopy. Table S5: Pearson and Kendall coefficients of pollen grain metric variables and classes/indices from the first two ordination axes of the principal component analysis (PCA) of *Vernonia* species—polar axis [PA], equatorial axis [EA], equatorial axis in polar view [PV], colporus length [CL], colporus width [CW], endoaperture length [EL], endoaperture width [EW], endoaperture class [Ecl], shape class [P/E = PA/EA]. Figure S1: Dendrogram built from the cluster analysis (Euclidean distance) of *Vernonia* specimens, similarity information remaining (%); Table S6: Characters and character states of pollen types *sensu* Jones (1981) updated according to the recent pollen terminology. 0—Sculpture type: echinate [0], subechinolophate [1], echinolophate [2], lophate [3]; 1—Aperture type: tricolporate [0], triporate [1]; 2—Tectum type 1: tectate perforate (Micropunctate) [0], semitectate [1], discontinuous* [2]; 3—Spine’s position 1: ridges [0], muri [1]; 4—Endoaperture type 1: elongated [0], 1; 5—Apertural Lacunae (Germinal furrows): separated by the poles by coincident muri [0], distinguishing features absent [1]; 6—Spine size: reduced [0], pronounced [1]; 7—Lacunae form: irregularly [0], regularly [1]; 8—Polar Lacunae: present [0], absent [1]; 9—Paired poral lacunae: present [0], absent [1]; 10—Apertural Lacunae 2: surrounded by a ridge [0], interrupted biparted muri [1]; 11—Muri type: elevated geometrically supported by conspicuous columellae [0], 1; ?—missing data. *—discontinuous tectum could also be a semitectate or tectate perforate tectum; 1 Jones (1981) does not present a second character state for this character.

## Abbreviations

LM	Light microscopy
SEM	Scanning electron microscopy
PCA	Principal component analysis
PA	Polar axis
EA	Equatorial axis
PV	Polar view
CL	Colporus length
CW	Colporus width
EL	Endoaperture length
EW	Endoaperture width
Ecl	Endoaperture class
P/E	Shape class
NSFDEB	National Science Foundation Division of Environmental Biology
NERC	Natural Environment Research Council
K	Royal Botanic Gardens Kew Herbarium
P	Muséum national d’histoire naturelle
X	Average
Sx	Average standard deviation
S	Sample standard deviation
V%	Coefficient of variability
CI	95% confidence interval
nm	Nanometres
Lab	Laboratory
LMD	Light Microdissection Microscope
kV	Kilovoltage

## Appendix A. Specimens Investigated

*Vernonia* Schreb.

*Species epithets* Authors. Country: Province. Collector and number, day Month year (Herbarium barcode, and short name) asterisk [*], only for SEM images (KMIC- slide barcode).

*Vernonia alleizettei* Humbert. Madagascar: Marojejy. H. Humbert 31770, 9 November–2 December 1959 (K 002545825, all25)—(1.671.001.672).

*Vernonia ambrensis* Humbert. Madagascar: Diego-Suarez/Antsiranana. L. Nusbaumer, P Ranirison LN 1555, 16 March 2005 (K 002545653, amb53)—(1.675.001.676).

*Vernonia ampandrandavensis* Humbert. Madagascar: Fianarantsoa. S. Malcomber et al. 1619, 22 September 1992 (K 002545655, amp55)—(1.677.001.678). Madagascar: Ranomafana. G.E. Schatz, J.S. Miller 2441, 5 December 1998 (K 002545657, amp57)—(1.679.001.680)

*Vernonia andapensis* Humbert. Madagascar: Andapa/Andembinibony. H. Humbert, R. Capuron 21940, 1 May 1905 (P 00061274, and74)—(1.681.001.682).

*Vernonia betonicifolia* Baker. Madagascar: Mangoky. H. Humbert, C.F. Swingle 4945, 29 July 1928 (K 002545580, beto80)—(1.683.001.684).

*Vernonia betsimisaraka* Humbert. Madagascar: Brickaville/Maroserana. G. Cours 4784, 27 November 1953 (P 00433928, bets28)—(1.685.001.686).

*Vernonia bojeri* Less. Madagascar. H Perrier de la Bathie 3255, s.d. (K 002545589, boj89)—(1.687.001.688)

*Vernonia carnotiana* Humbert. Madagascar: Toliara, Eminiminy. B. Randriamampionona 372, 4–24 May 1993 (K 002545704, car04)—(1.689.001.690). Madagascar: Antsiranana. A, Randrianasolo 28, 12 May 1989. (K 002545706, car06)—(1.691.001.692).

*Vernonia cephalophora* Humbert. Madagascar:Ambalabao. R. Capuron 29221—SF, 7 June 19721. (K 002545660, cep60), (1.693.001.694). Madagascar: Antsiranana. L.J. Razafitsalama et al. 706, 1 July 2005 (K 000546025, cep25)—(1.695.001.696).

*Vernonia decaryana* Humbert. Madagascar: Ambovombe. R. Decary 2799, 1 June 1924 (P 00346022, dec22)—(1.697.001.698).

*Vernonia diversifolia* Bojer ex DC. subsp. *diversifolia.* Madagascar: Fianarantsoa. H. Ralimanana et al. RLI 1479, 4 May 2010 (K 000663263, div63)—(1.699.001.700). Madagascar: Toliara. B. Randriamampionona 697, 7 April 1994 (K 000662137, div37)—(1.701.001.702).

*Vernonia diversifolia* subsp. *ikopae* (Drake) Humbert. Madagascar: Besinkara. L. Gautier, C Chatelain LG 2757, 21 May 1995 (K 002545577, div77)—(1.703.001.704). Madagascar: Diego-Suarez/Antsiranana. P. Ranirison PR 637, 14 April 2004 (K 002545576, div76)—(1.705.001.706).

*Vernonia homolleae* Humbert. Madagascar: Beampingaratra. H. Humbert 6453, 6–7 November 1928 (K 002545820, hom20)—(1.707.001.708)

*Vernonia humillima* Humbert. Madagascar: Itremo (West Betsileo). H. Humbert 30129. 8 May 1905 (P 00433978) *—(1.709.001.710).

*Vernonia ikongensis* Humbert. Madagascar: Ikongo/Farafangana. R. Decary 5530. 12 October 1926 (P 00433987, iko87)—(1.711.001.712).

*Vernonia isalensis* Humbert. Madagascar. H. Humbert 2855, 20–22 October 1921 (K 002545658, isa58)—(1.713.001.714).

*Vernonia kenteocephala* Baker. Madagascar: Ankarafantsika. B. du Puy et al. MB 125, 16 March 1990 (K 002545732, ken32)—(1.715.001.716).

*Vernonia latisquamata* (Humbert) Humbert. Madagascar: Diego-Suarez/Antsiranana. N.S. Rosoavaivo, A.J. tahinarivony NSR067, 12 June 2012 (K 002545669, lat69)—(1.717.001.718).

*Vernonia leandrii* Humbert. Madagascar: Majunga/Mahajanga. L. Ranaivoarisoa, I. Luino, LUR 031, 7 March 2020 (K 002545654, lea54)—(1.719.001.720).

*Vernonia lemurica* Humbert. Madagascar: Diego-Suarez/Antsiranana. L. Nusbaumer, P. Ranirison LN 1582, 11 December 2005 (K 002545852, lem52)—(1.721.001.722).

*Vernonia mandrarensis* Humbert. Madagascar: Toliara. F. Ratovoson 1445, 29 February 2008 (K 002545591, mand91)—(1.723.001.724).

*Vernonia manongarivensis* Humbert. Madagascar: Antananarivo. H.H. Schmidt et al. 4294, 22 November 2003 (K 000438767, mano67)—(1.725.001.726).

*Vernonia mecistophylla* Baker. Madagascar: Besinkara. L. Gautier, C. Chatelain LG 2318, 14 June 1994 (K 002545667, mec 67)—(1.727.001.728).

*Vernonia monantha* Humbert. Madagascar: Diego-Suarez/Antsiranana. L. Gautier et al. LG3295, 6 January 1998 (K 002525869, mon69)—(1.729.001.730).

*Vernonia neocoursiana* Humbert. Madagascar: Taomasina. H. van de Werff et al. 13635, 3 November 1994 (K 002545673, neoc79)—(1.731.001.732). Madagascar: Alaotra Didy, Ambodinanto. G. Cours 4700 (P 00434044, neoc44)—(1.733.001.734).

*Vernonia neoperrieriana* Humbert. Madagascar: Toliara. P.B. Phillipson et al. 5896, 15 March 2006 (K 002545581, neop81)—(1.735.001.736).

*Vernonia pachyclada* Baker. Madagascar: Toamasina. P. Antilahimena et al. 4026, 18 October 2005 (K 002545676, pac76)—(1.737.001.738). Madagascar: J.M. Hidelbrandt et al. 3635, November 1880 (K 002545680, pac80)—(1.739.001.740). Madagascar: Toamasina. A. Razanatsima et al. 428, 22 September 2005 (K 002545674, pac74)—(1.741.001.742).

*Vernonia pellegrinii* Humbert. Madagascar: Isalo. H. Poisson 547, 12 August 1922 (P 00434060, pel60)—(1.743.001.744).

*Vernonia platylepis* Drake. Madagascar: Antsiranana. J.B. Leopold et al. 29, 14 May 2005 (K 002545740, pla40)—(1.745.001.746).

*Vernonia pseudoappendiculata* Humbert. Madagascar: Bekolosky. L. Gautier, C Chatelain LG 2332, 15 June 1994 (K 002545860, pse60)—(1.747.001.748).

*Vernonia sakalava* Humbert. Madagascar: Mahavavy (Ambongo). H. Perrier de la Bâthie 1448, 1 May 1902 (P 00435018, sak18)—(1.749.001.750).

*Vernonia sambiranensis* Humbert. Madagascar: Sambirano/Manongarivo. H. Perrier de la Bathie 3209, 1 May 1909 (P 00435022, sam23)—(1.751.001.752).

*Vernonia seyrigii* Humbert. Madagascar: Ambovombe. R. Decary 8577, 12 March 1931 (P 0043502, sey29)—(1.753.001.754).

*Vernonia speiracephala* Baker. Madagascar: Toamasina. H. Razanatsoa, R. Razafindsay 442 10 January 2005 (K 002545754) *—(1.755.001.756). Madagascar: Toamasina. P. Antilahimena, F. Edmond 3991, 10 December 2005 (K 002545755, spe55)—(1.757.001.758).

*Vernonia tanalensis* Baker. Madagascar: Andringitra. H. Humbert 3774, 27 November/8 December 1924 (K 002545450, tan50)—(1.759.001.760).

*Vernonia tropophila* Humbert. Madagascar: Majunga/Mahajanga. L. Gautier, I. Luino, A.P. Rasolonjatovo LG 6190, 22 November 2015 (P 00945370, tro70)—(1.761.001.762).

*Vernonia vohemarensis* Humbert. Madagascar:Vohemaro/Dareno. H. Perrier de la Bâthie 3151, s.d. (P 00435070, voh70)—(1.763.001.764).
