# Supplementary material for: New Pollen Morphological Perspectives into Vernonia (Compositae—Vernonieae) from Madagascar
Source: Plants (Basel). 2026 Jun 22;15(12):1927. doi: 10.3390/plants15121927 (PMC13306231; doi:10.3390/plants15121927)
Supplement: Supplementary file 1 [file plants-15-01927-s001.zip › Supplementary Material Table S1.pdf]

Specimen	Equatorial Axis (EV)			Polar Axis (EV)			P/E	Equatorial Axis (PV)			Size
	CI - ($x \pm sx$) CI +	s	V%	CI - ($x \pm sx$) CI +	s	V%	x	CI - ($x \pm sx$) CI +	s	V%	CI
all 25	40.9 (41.6 ± 0.4) 42.4	1.8	4.4	33.9 (34.5 ± 0.3) 35.1	1.5	4.3	0.8	39.1 (39.9 ± 0.4) 40.7	1.9	4.8	M
amb 53	54.2 (54.9 ± 0.3) 55.5	1.6	2.9	51.3 (52.1 ± 0.3) 52.8	1.7	3.3	0.9	54.7 (55.7 ± 0.5) 56.8	2.5	4.8	L
amp 55	39.0 (38.9 ± 0.4) 40.7	2.1	5.2	34.9 (35.2 ± 0.3) 35.9	1.6	4.6	0.9	38.5 (39.4 ± 0.5) 40.4	2.3	5.8	M
amp 57	35.0 (35.7 ± 0.3) 36.4	1.6	4.6	30.9 (31.8 ± 0.4) 32.7	2.1	6.7	0.9	35.5 (36.8 ± 0.6) 38.0	3.0	8.7	M
and 74	37.6 (38.1 ± 0.2) 38.6	1.6	4.1	33.9 (34.4 ± 0.2) 35.0	1.7	5.0	0.9	33.1 (33.3 ± 0.1) 33.5	0.8	2.4	M
beto 80	32.4 (33.2 ± 0.3) 33.9	1.7	5.2	31.4 (32.1 ± 0.4) 32.9	1.8	5.7	1.0	33.8 (34.3 ± 0.2) 34.7	1.2	3.4	M
bets 28	41.1 (41.7 ± 0.3) 42.3	1.4	3.3	36.3 (36.9 ± 0.3) 37.5	1.4	3.9	0.9	38.7 (39.5 ± 0.4) 40.7	2.0	5.0	M
boj 89	47.9 (48.7 ± 0.4) 49.5	1.9	3.9	43.7 (47.5 ± 0.4) 48.3	2.0	4.2	1.0	48.4 (49.1 ± 0.3) 49.7	1.5	3.1	M
car 04	39.8 (40.5 ± 0.4) 41.2	1.8	4.4	35.8 (36.4 ± 0.3) 37.0	1.4	3.8	0.9	38.6 (39.2 ± 0.3) 39.8	1.5	3.7	M
car 06	35.9 (36.5 ± 0.3) 37.1	1.8	4.8	31.9 (32.6 ± 0.3) 33.3	1.8	5.7	0.9	34.8 (35.3 ± 0.3) 35.9	2.4	6.7	M
cep 25	68.8 (69.8 ± 0.5) 70.8	2.5	3.6	69.4 (70.3 ± 0.4) 71.3	2.2	3.2	1.0	68.1 (69.1 ± 0.5) 70.2	2.5	3.7	L
cep 60	60.7 (62.1 ± 0.7) 63.3	3.5	5.7	59.9 (61.1 ± 0.6) 62.4	3.0	4.9	1.0	56.5 (58.4 ± 0.9) 60.2	4.4	7.5	L
dec 22	36.7 (37.1 ± 0.2) 37.5	0.9	2.3	33.3 (34.1 ± 0.2) 34.5	1.0	2.9	0.9	34.5 (35.9 ± 0.3) 36.5	1.3	3.6	M
div 37	37.2 (37.8 ± 0.3) 38.4	1.4	3.7	36.8 (37.3 ± 0.3) 37.8	1.3	3.4	1.0	36.2 (36.8 ± 0.3) 37.3	1.3	3.6	M
div 63	33.7 (34.6 ± 0.4) 35.5	2.1	6.1	32.2 (33.1 ± 0.4) 34.0	2.2	6.5	1.0	34.1 (34.9 ± 0.4) 35.6	1.9	5.5	M
div 76	34.5 (35.2 ± 0.3) 35.9	1.7	4.9	31.7 (32.3 ± 0.3) 32.8	1.3	4.0	0.9	34.4 (35.2 ± 0.4) 36.0	1.9	5.4	M
div 77	33.4 (33.9 ± 0.2) 34.3	1.1	3.2	31.2 (31.6 ± 0.2) 32.0	0.9	3.0	0.9	33.4 (33.9 ± 0.3) 34.5	1.4	4.2	M
hom 20	40.9 (41.4 ± 0.3) 42,0	1.4	3.3	37.0 (37.8 ± 0.4) 38.5	1.8	4.7	0.9	38.4 (39.4 ± 0.5) 40.3	2.3	5.7	M
iko 87	41.2 (41.6 ± 0.2) 42.1	1.2	2.8	38.7 (39.2 ± 0.2) 39.7	1.2	3.0	0.9	40.2 (40.9 ± 0.3) 41.6	1.7	4.2	M
isa 58	42.2 (43.1 ± 0.4) 43.9	2.1	4.9	43.9 (44.5 ± 0.3) 45.0	1.7	3.7	1.0	45.6 (46.2 ± 0.3) 46.8	1.5	3.2	M
ken 32	56.6 (57.6 ± 0.5) 58.6	2.4	4.2	53.9 (54.8 ± 0.5) 55.8	2.4	4.3	1.0	55.6 (56.5 ± 0.4) 57.6	2.0	3.6	L
lat 69	69.4 (70.0 ± 0.7) 72.0	3.3	4.6	69.8 (71.0 ± 0.6) 72.1	2.8	3.9	0.9	69.7 (70.6 ± 0.6) 72.2	3.1	4.3	L
lea 54	39.7 (40.7 ± 0.5) 41.7	2.5	6.1	35.0 (36.1 ± 0.5) 37.2	2.6	7.2	0.9	40.4 (41.3 ± 0.5) 42.3	2.4	5.8	M
lem 52	38.0 (38.7 ± 0.3) 39.3	1.6	4.1	35.1 (35.6 ± 0.2) 36.1	1.1	3.2	0.9	36.0 (36.6 ± 0.3) 37.1	1.4	3.9	M
mand 91	44.2 (45.2 ± 0.5) 46.2	2.4	5.3	41.8 (42.5 ± 0.2) 43.2	1.7	3.9	0.9	45.6 (46.3 ± 0.4) 47.1	1.9	4.0	M
mano 67	43.1 (43.9 ± 0.4) 44.7	2.0	4.4	40.8 (41.6 ± 0.4) 42.0	1.9	4.5	0.9	41.6 (42.3 ± 0.3) 43.0	1.7	3.9	M

mec 67	64.5 (65.9 ± 0.7) 67.3	3.4	5.1	58.2 (59.9 ± 0.8) 61.5	4.0	6.7	0.9	64.1 (65.5 ± 0.7) 66.8	3.3	5.0	M
mon 69	40.1 (40.5 ± 0.2) 41.0	1.6	4.0	38.0 (38.6 ± 0.3) 39.2	1.9	4.9	1.0	38.1 (38.6 ± 0.2) 39.0	1.3	3.3	M
neoc 44	39.2 (39.9 ± 0.3) 40.6	1.7	4.2	34.9 (35.5 ± 0.3) 36.1	1.5	4.1	0.9	38.0 (38.7 ± 0.3) 39.3	1.6	4.3	M
neoc 79	37.6 (38.6 ± 0.5) 39.6	2.3	6.8	33.2 (34.1 ± 0.5) 35.1	2.3	6.8	0.9	35.1 (36.4 ± 0.6) 37.8	3.2	8.9	M
neop 81	40.3 (40.9 ± 0.3) 41.4	1.3	3.3	39.9 (40.4 ± 0.3) 41.0	1.3	3.3	0.9	39.9 (40.7 ± 0.4) 41.4	1.8	4.5	M
pac 74	43.3 (44.1 ± 0.9) 44.8	1.9	4.3	40.4 (41.0 ± 0.3) 41.6	1.4	3.5	0.9	43.6 (44.2 ± 0.3) 44.8	1.4	3.1	M
pac 76	41.1 (42.1 ± 0.5) 43.4	2.4	5.6	35.4 (36.0 ± 0.3) 36.6	1.5	4.3	0.9	40.7 (41.2 ± 0.2) 41.6	1.1	2.7	M
pac 80	44.3 (45.2 ± 0.4) 46.1	2.2	4.9	39.8 (40.7 ± 0.4) 41.5	2.0	4.9	0.9	44.6 (45.3 ± 0.4) 46.0	1.8	3.8	M
pel 60	43.8 (44.5 ± 0.3) 45.2	1.7	3.9	39.0 (39.8 ± 0.4) 40.6	1.9	4.7	0.9	42.7 (43.3 ± 0.3) 43.9	1.4	3.3	M
pla 40	50.2 (70.9 ± 0.3) 51.3	1.7	3.4	47.8 (47.9 ± 0.5) 48.9	2.3	4.9	0.9	49.9 (50.5 ± 0.3) 51.1	1.5	3.0	M-L
pse 60	41.9 (42.6 ± 0.3) 43.2	1.5	3.6	37.3 (38.1 ± 0.3) 38.8	1.7	4.6	0.9	40.7 (41.4 ± 0.3) 42.1	1.7	4.1	M
sak 18	41.4 (42.1 ± 0.3) 42.8	1.6	3.9	37.8 (38.3 ± 0.3) 38.8	1.3	3.4	0.9	40.3 (41.0 ± 0.3) 41.7	1.7	4.2	M
sam 23	42.3 (43.2 ± 0.5) 44.2	2.3	5.4	38.0 (38.8 ± 0.4) 39.6	1.9	4.9	0.9	41.3 (42.0 ± 0.4) 42.2	1.9	4.5	M
sey 29	47.9 (48.3 ± 0.3) 49.0	1.4	3.9	42.0 (42.8 ± 0.3) 43.5	1.7	4.0	0.9	46.3 (47.0 ± 0.4) 47.8	1.9	4.0	M
spe 55	38.5 (39.0 ± 0.2) 39.4	1.1	2.9	35.0 (35.7 ± 0.3) 36.6	1.7	4.8	0.9	36.9 (37.5 ± 0.3) 38.2	1.5	4.0	M
tal 50	41.5 (42.1 ± 0.3) 42.7	1.5	3.5	36.8 (37.5 ± 0.4) 38.3	1.7	4.7	0.9	39.1 (39.9 ± 0.4) 40.8	2.0	5.0	M
tro 70	38.0 (38.8 ± 0.4) 39.7	2.1	5.5	35.1 (36.1 ± 0.5) 37.1	2.4	6.8	0.9	37.2 (37.9 ± 0.4) 38.7	1.8	4.8	M
voh 70	53.9 (55.0 ± 0.5) 56.0	2.5	4.6	50.7 (51.7 ± 0.5) 52.8	2.5	4.9	0.9	52.8 (53.8 ± 0.5) 54.8	2.4	4.4	L

Supplementary Material Table S1. *Vernonia* dimensions (µm) pollen grains in equatorial and polar view using light microscopy.

Note. Specimens were identified by the abbreviation of the epithet and the last two numbers of the herbarium voucher (see Specimen analysed). Equatorial View (EV), polar view (PV), Confidence Interval (CI) at 95% of probability of the lowest sample values (IC-) and highest sample values (IC+), arithmetic mean (*x*), average standard deviation (*sx*), sample standard deviation (*s*), coefficient of variability (V%), oblate spheroidal (OS).
