# Supplementary material for: New Pollen Morphological Perspectives into Vernonia (Compositae—Vernonieae) from Madagascar
Source: Plants (Basel). 2026 Jun 22;15(12):1927. doi: 10.3390/plants15121927 (PMC13306231; doi:10.3390/plants15121927)
Supplement: Supplementary file 1 [file plants-15-01927-s001.zip › Supplementary Material Table S2.pdf]

Specimen	Lacunae	Colporus	Endoaperture	Ornamentation		Exine
	L - W	L - W	W (W/C) L	Spine	Lo - Mu	S (S/N) N - E
all 25	--	26.0 – 5.0	8.7 (1.8) 5.0	3.8	--	4.4 (2.0) 2.2 – 6.6
amb 53	26.4 – 11.7	14.4 – 4.1	10.0 (1.3) 7.9	--	11.7 – 2.9	9.3 (3.8) 2.5 – 11.9
amp 55	--	23.0 – 6.2	8.9 (1.6) 6.0	2.4	--	3.6 (1.7) 2.0 – 5.6
amp 57	--	21.9 – 4.8	4.4 (1.7) 7.3	2.4	--	4.6 (2.0) 2.3 – 6.9
and 74	--	24.7 – 6.1	7.3 (1.9) 4.0	3.0		4.1 (1.8) 2.2 – 6.3
beto 80	--	16.1 – 3.7	4.6 (1.8) 2.5	2.1	--	3.4 (2.1) 1.6 – 5.0
bets 28	--	27.7 – 5.2	7.4 (1.8) 4.3	3.2	--	4.1 (1.5) 2.7 – 6.8
boj 89	20.8 – 9.0	8.9 – 3.9	6.2 (0.8) 7.7	--	9.6 – 2.7	7.0 (3.5) 2.0 – 9.0
car 04	--	26.0 – 3.8	8.8 (2.0) 4.5	2.6	--	4.1 (1.8) 2.2 – 6.4
car 06	--	22.7 – 3.7	7.5 (1.8) 4.3	2.7	--	3.5 (1.9) 1.8 – 5.3
cep 25	25.5 – 13.6	12.9 – 9.8	13.8 (1.0) 13.5	--	16.6 – 3.7	12.7 (4.5) 3.0 – 15.6
cep 60	21.8 – 12.6	13.7 – 8.0	11.5 (1.0) 11.1	--	15.0 – 3.6	9.0 (3.6) 2.6 – 11.6
dec 22	--	23.2 – 4.9	6.9 (1.9) 3.6	2.8	--	3.6 (1.5) 2.4 – 6.0
div 37	--	23.3 – 6.8	6.9 (1.0) 6.9	2.2	--	3.2 (1.8) 1.8 – 5.0
div 63	--	23.6 – 4.3	6.3 (1.0) 6.1	2.3	--	3.1 (2.2) 1.5 – 4.6
div 76	--	23.4 – 5.0	4.8 (1.0) 5.2	2.9	--	3.0 (1.8) 1.7 – 4.8
div 77	--	23.7 – 3.2	4.7 (1.4) 3.5	2.9	--	3.2 (2.2) 1.5 – 4.7
hom 20	--	28.4 – 3.7	7.0 (2.5) 2.9	3.0	--	4.1 (1.8) 2.3 – 6.4
iko 87	--	27.9 – 6.1	9.1 (2.4) 3.8	2.2	--	4.1 (1.8) 2.2 – 6.3
isa 58	--	22.8 – 5.9	7.1 (1.3) 5.5	3.3	--	4.8 (3.0) 1.6 – 6.5
ken 32	23.2 – 8.9	14.1 – 4.8	9.1 (1.2) 8.0	--	13.1 – 2.8	9.1 (3.3) 2.8 – 11.9
lat 69	27.4 – 14.5	15.6 – 11.5	14.6 (0.8) 19.2	--	19.7 – 3.2	11.7 (5.1) 2.4 – 14.1
lea 54	--	25.3 – 6.2	6.6 (1.2) 5.9	2.7	--	4.9 (2.1) 2.3 – 7.2
lem 52	--	25.7 – 7.1	9.6 (1.6) 6.2	2.5	--	4.4 (1.6) 2.1 – 6.5
mand 91	--	27.9 – 6.3	9.4 (1.4) 6.9	2.6	--	5.5 (2.5) 2.3 – 7.8
mano 67	--	32.6 – 6.8	10.6 (1.9) 5.7	2.4	--	3.6 (1.8) 2.0 – 6.5
mec 67	20.9 – 10.5	9.7 – 6.2	9.4 (0.9) 10.5	--	13.9 – 3.0	10.9 (4.8) 2.3 – 13.2
mon 69	--	24.9 – 4.1	8.4 (2.4) 3.6	2.3	--	3.6 (2.0) 1.7 – 5.3
neoc 44	--	26.5 – 4.5	6.5 (1.9) 3.4	2.7	--	3.3 (1.8) 1.7 – 5.0
neoc 79	--	23.8 – 5.4	8.6 (1.6) 5.6	2.6	--	4.1 (2.0) 2.1 – 6.2
neop 81	10.9 – 7.5	7.9 – 4.5	6.8 (1.0) 6.6	1.5	10.2 – 3.4	5.2 (2.6) 2.1 – 7.2
pac 74	--	28.1 – 6.1	8.0 (1.2) 6.5	3.4	--	4.0 (2.1) 2.0 – 6.0
pac 76	--	25.9 – 5.7	6.4 (1.9) 3.4	3.5	--	3.9 (2.1) 1.9 – 5.8
pac 80		29.1 – 6.5	9.0 (1.4) 6.3	3.5	--	4.6 (2.7) 1.7 – 6.4
pel 60	--	27.6 – 5.7	8.7 (2.5) 3.6	3.8	--	4.2 (1.8) 2.4 – 6.6

pla 40	24.6 – 8.4	15.5 – 6.5	9.2 (1.0) 9.0	--	10.4 – 2.6	8.4 (3.6) 2.4 – 10.7
pse 60	--	28.7 – 5.1	9.2 (2.2) 4.2	3.1	--	4.5 (2.7) 2.2 – 6.8
sak 18	--	26.7 – 6.7	8.7 (1.5) 5.9	3.4	--	4.4 (2.0) 2.1 – 6.5
sam 23	--	28.4 – 7.2	9.2 (1.5) 6.1	2.6	--	4.2 (1.9) 2.2 – 6.4
sey 29	--	31.3 – 6.3	8.4 (1.5) 5.7	2.6	--	5.1 (2.5) 2.1 – 7.2
spe 55	--	25.8 – 4.7	9.4 (2.5) 3.8	3.1	--	3.9 (1.6) 2.0 – 5.9
tal 50	--	26.0 – 4.9	7.9 (2.7) 3.0	2.7	--	5.0 (1.7) 2.9 – 7.9
tro 70	15.7 – 7.6	6.7 – 4.2	6.5 (1.4) 4.8	--	7.5 – 1.9	4.0 (2.6) 1.6 – 5.6
voh 70	20.3 – 7.6	7.6 – 4.9	6.5 (1.4) 4.7	--	10.5 – 2.6	9.8 (4.4) 2.3 – 12.1

Supplementary Material Table S2. Dimensions (μm) pollen grains aperture, spine, reticulum and exine layers in equatorial view using light microscopy.

Note. Specimens were identified by the abbreviation of the epithet and the last two numbers of the herbarium voucher (see Specimen analysed). Length (L), Width (W), Width/Length (W/L), Lophae (Lo), Muri (M), Sexine (S), Sexine/Nexine (S/N), Nexine (N), Exine (E).
