# Supplementary material for: New Pollen Morphological Perspectives into Vernonia (Compositae—Vernonieae) from Madagascar
Source: Plants (Basel). 2026 Jun 22;15(12):1927. doi: 10.3390/plants15121927 (PMC13306231; doi:10.3390/plants15121927)
Supplement: Supplementary file 1 [file plants-15-01927-s001.zip › Supplementary Material Table S3.pdf]

Supplementary Material Table S3: Morphology and ultrasculpture of *Vernonia* pollen grains Type I (*Sublophate*) using light and scanning electron microscopy.

Specie	Subtype	PA	Colporus size, apices	Endoaperture class, terminals	Lophae	Spine apices	*Lacunae/Sublacunae
V. alleizettei	a	S	L, pointed	Very lalongate, acute	Irregual, nano- to microreticulate	Acute	Nano- to microreticulate
V. ampandranadavensis	a	S	L, pointed	Very lalongate, rounded	Irregular, nanoreticulate	Acute	Nanoreticulate
V. andapensis	a	VS	L, pointed	Very lalongate, rounded	Irregular, nanoreticulate	Acute	Nanoreticulate
V. betonicifolia	a	VS	VL, pointed	Very lalongate, rounded	Polygonal, nanoreticulate	Acute	Nanoreticulate
V. betsimisaraka	a	VS	VL, rounded	Very lalongate, acute	Irregular, nano- to microreticulate	Acute	Nano- to microreticulate
V. carnotiana	a	VS	VL, rounded	Very lalongate, acute	Irregular, nanoreticulate-perforate	Acute	Nanoreticulate-perforate
V. decaryana	a	M	M, rounded	Very lalongate, acute	Polygonal, nano- to microreticulate-perforate	Acute	Nano- to microreticulate-perforate
V. diversifolia	a	M	M, pointed	Circular to lalongate, acute	Irregular, nanoreticulate-perforate	Acute	Nanoreticulate-perforate
V. diversifolia subsp. *ikopae*	a	S	L, pointed	Circular to lalongate, acute	Polygonal, nanoreticulate-perforate	Acute	Nanoreticulate-perforate
V. homolleae	a	VS	L, pointed	Very lalongate, acute	Irregular, nanoreticulate-perforate	Acute	Nanoreticulate-perforate
V. humillima	a	S	L, pointed	--	Irregular, nanoreticulate-perforate	Acute to rounded	Nanoreticulate-perforate
V. ikongensis	a	S	L, pointed	Very lalongate, acute	Irregular, nanoreticulate-perforate	Rounded	Nanoreticulate-perforate
V. isalensis	a	M	M, pointed	Circular to lalongate, rounded	Polygonal, nanoreticulate-perforate with nanospines	Rounded	Nanoreticulate-perforate
V. leandrii	a	S	L, pointed	Circular to lalongate, acute	Irregular, nanoreticulate-perforate	Rounded	Nanoreticulate-perforate
V. lemurica	a	S	L, rounded	Lalongate to very lalongate, rounded	Irregular, nanoreticulate-perforate	Rounded	Nanoreticulate-perforate
V. mandrarensis	b	VS	VL, rounded	Lalongate to very lalongate, rounded	Irregular, nano- to microreticulate-perforate	Rounded	Granulate-perforate with incomplete nanoreticulum
V. manongarivensis	a	S	L, rounded	Lalongate to very lalongate, acute	Irregular, nanoreticulate-perforate	Rounded	Nanoreticulate-perforate
V. monantha	a	S	L, rounded	Lalongate to very lalongate, acute	Irregular, nanoreticulate-perforate	Rounded	Perforate
V. neocoursiana	a	VS	VL, pointed	Very lalongate, acute	Irregular, nanoreticulate-perforate	Acute	Nanoreticulate-perforate

V. pachyclada	a	S	L, pointed	Lalongate to very lalongate, rounded	Irregular, nanoreticulate-perforate	Acute	Nanoreticulate-perforate
V. pellegrinii	a	S	L, pointed	Very lalongate, rounded	Polygonal, nano- to microreticulate-perforate	Rounded	Nanoreticulate-perforate
V. pseudoappendiculata	a	S	L, pointed	Very lalongate, acute	Irregular, nanoreticulate-perforate with nanospines	Rounded	Nanoreticulate-perforate
V. sakalava	a	M	M, rounded	Lalongate to very lalongate, rounded	Polygonal, nanoreticulate-perforate with nanospines	Acute	Nanoreticulate-perforate
V. sambiranensis	b	VS	VL, rounded	Lalongate to very lalongate, rounded	Polygonal, nano- to microreticulate-perforate	Acute	Granulate-perforate with incomplete nanoreticulum
V. seyrigii	b	VS	VL, rounded	Lalongate to very lalongate, rounded	Polygonal, nano- to microreticulate-perforate	Rounded	Granulate-perforate with incomplete nanoreticulum
V. speiracephala	a	VS	VL, rounded	Very lalongate, rounded	Irregular, nanoreticulate-perforate	Acute	Nanoreticulate-perforate
V. tanalensis	a	VS	VL, rounded	Very lalongate, acute	Irregular, nanoreticulate-perforate	Acute	Nanoreticulate-perforate

Notes: Polar Area (PA), Very Small (VS), Small (S), Medium (M), Large (L), Very Large (VL), Subtype a (a), Subtype b (b).

Subtype a (a), Subtype b (b), Present only in type b (*).
