# Supplementary material for: New Pollen Morphological Perspectives into Vernonia (Compositae—Vernonieae) from Madagascar
Source: Plants (Basel). 2026 Jun 22;15(12):1927. doi: 10.3390/plants15121927 (PMC13306231; doi:10.3390/plants15121927)
Supplement: Supplementary file 1 [file plants-15-01927-s001.zip › Supplementary Material Table S4.pdf]

Supplementary Material Table S4: Morphology and ultrasculpture of *Vernonia* pollen grains Type II (*Lophate*) using light and scanning electron microscopy.

Species	Size, subtype	PA	Lacunae size, constriction, ridges	Colporus size, apices	Endoaperture class	Ornamentation, lopha	Lacunae; columellae
V. ambrensis	L, a	L	S, Ps, Ps	S, slightly acute	Lalongate	Echinilophate, nano- to microechinate	Smooth; simple to digitate, smooth
V. bojeri	M – L, a	L	S, Ps, Ab	S, rounded	Lolongate	Psilolophate, perforate-granulate	Granulate^{-}; digitate, smooth
V. cephalophora	L, a	L	S, Ps. Ab	S, rounded	Circular to lalongate	Psilolophate, nano- to microrugulate-granulate	Granulate, digitate, granulate
V. kenteocephala	L, a	L	M, Ps, Ab	S, slightly acute	Circular to lalongate	Psilolophate, perforate^{-}, granulate^{-}	Granulate^{-}; digitate, smooth
V. latisquamata	L, a	VL	VS, Ab, Ab	VS, rounded	Lolongate	Psilolophate, perforate-granulate	Granulate^{-}; digitate, granulate^{-}
V. mecistophylla	L, a	L	S, Ps, Ab	VS, rounded	Circular to lolongate	Psilolophate, perforate^{-}, granulate^{-}	Granulate^{-}; digitate, granulate^{-}
V. neoperrieriana	M. b	VL	VS, Ab, Ab	VS, pointed	Circular to lalongate	Echinolophate, nanoreticulate	Granulate-nanorugulate^{-}; non-visible
V. platylepsis	M – L, a	L	M, Ab, Ps	S, rounded	Circular to lalongate	Psilolophate, perforate^{-}, granulate^{-}	Granulate^{-}; digitate, granulate^{-}
V. tropophila	M, a	L	S, Ab, Ab	S. rounded	Circular to very lalongate	Echinolophate, nano- to microechinate, perforate^{-}, granulate^{-}	Granulate^{-}; simple to digitate, granulate^{-}
V. vohemarensis	L, a	L	S, Ps, Ps	S, rounded	Very lalongate	Psilolophate, perforate^{-}, granulate^{-}	Granulate^{-}; simple to digitate, granulate^{-}

Notes: Polar Area (PA), Very Small (VS), Small (S), Medium (M), Large (L), Very Large (VL), Present (Ps), Absent (Ab), Sparse ($^{-}$), Subtype a (a), Subtype b (b).
