# Supplementary material for: New Pollen Morphological Perspectives into Vernonia (Compositae—Vernonieae) from Madagascar
Source: Plants (Basel). 2026 Jun 22;15(12):1927. doi: 10.3390/plants15121927 (PMC13306231; doi:10.3390/plants15121927)
Supplement: Supplementary file 1 [file plants-15-01927-s001.zip › Supplementary Material Table S5.pdf]

Variable	Axis 1	Axis 2
EA	-0.2995	-0.0833
PA	-0.3229	-0.0613
PV	-0.2935	-0.0664
P/E	-0.0091	0.0119
CL	0.4304	-0.7966
CW	-0.2530	-0.3966
EW	-0.2352	-0.3742
EL	-0.5944	-0.1998
CEN	0.2489	-0.1140

Supplementary Material Table S5. Pearson and Kendall coefficients of pollen grain metric variables and classes/indices from the first two ordination axes of the principal component analysis (PCA) of *Vernonia* species. polar axis [PA], equatorial axis [EA], equatorial axis in polar view [PV], colporus length [CL], colporus width [CW], endoaperture length [EL], endoaperture width [EW], endoaperture class [Ecl], shape class [P/E = PA/EA].
