# Supplementary material for: New Pollen Morphological Perspectives into Vernonia (Compositae—Vernonieae) from Madagascar
Source: Plants (Basel). 2026 Jun 22;15(12):1927. doi: 10.3390/plants15121927 (PMC13306231; doi:10.3390/plants15121927)
Supplement: Supplementary file 1 [file plants-15-01927-s001.zip › Supplementary Material Table S6.pdf]

Characters	Sculpture type	Aperture Type	Tectum type	Spine’s position	Endoaperture type	Apertural lacunae	Spine’s size	Lacunae form	Polar lacunae	Paired polar lacunae	Apertural Lacunae	Muri type
	0	1	2	3	4	5	6	7	8	9	10	11
Pollen types						Character state						
Type A	0,1	0	0	0, 1	?	?	?	?	?	?	?	?
Type B	2	0	0, 2	0	0	0	0, 1	0, 1	0	0, 1	?	?
Type C	2	0	0, 2	1	?	0, 1	0, 1	1	1	0, 1	?	?
Type D	2	1	0, 2	1	?	?	0, 1	1	?	?	0	?
Type E	3, 1	?	1	?	?	1	?	0	?	?	?	0
Type F	3, 2	?	1	?	?	?	?	?	?	?	1	0

Supplementary Material Table S6: Characters and character states of pollen types *sensu* Jones (1981) updated according to the recent pollen terminology. 0 - Sculpture type: echinate [0], subechinolophate [1], echinolophate [2], lophate [3]; 1 - Aperture type: tricolporate [0], triporate [1]; 2 - Tectum type 1: tectate perforate (Micropunctate) [0], semitectate [1], discontinuous* [2]; 3 - Spine’s position 1: ridges [0], muri [1]; 4 - Endoaperture type 1: elongated [0], [1]; 5 - Apertural Lacunae (Germinal furrows): separated by the poles by coincident muri [0], distinguishing features absent [1]; 6 - Spine size: reduced [0], pronounced [1]; 7 - Lacunae form: irregularly [0], regularly [1]; 8 - Polar Lacunae: present [0], absent [1]; 9 - Paired poral lacunae: present [0], absent [1]; 10 - Apertural Lacunae 2: surrounded by a ridge [0], interrupted biparted muri [1]; 11 - Muri type: elevated geometrically supported by conspicuous columellae [0], [1]; ? - missing data. * - discontinuous tectum could also be a semitectate or tectate perforate tectum; [1] Jones (1981) does not present a second character state for this character.
