# Supplementary figures and images for: New Pollen Morphological Perspectives into Vernonia (Compositae—Vernonieae) from Madagascar
Source: Plants (Basel). 2026 Jun 22;15(12):1927. doi: 10.3390/plants15121927 (PMC13306231; doi:10.3390/plants15121927)

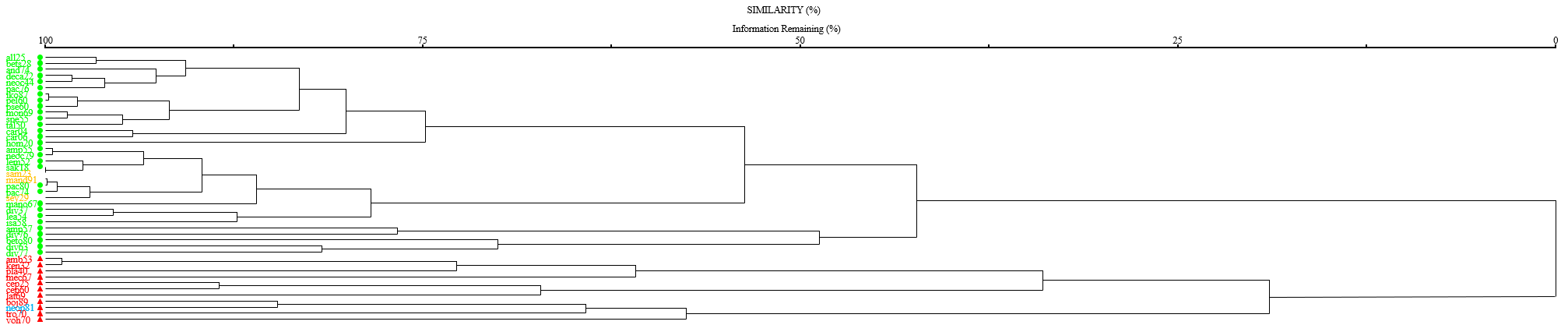

Supplement: Supplementary file 1 [file plants-15-01927-s001.zip › Supplementary Material Figure S1.png]
